# Adherence to iron and folic acid supplementation during pregnancy among postnatal mothers seeking maternal and child healthcare at Kakamega level 5 hospital in Kenya: a cross-sectional study

**DOI:** 10.12688/wellcomeopenres.16699.1

**Published:** 2021-04-12

**Authors:** Felix Bahati, Salome Kairu-Wanyoike, Japheth Mativo Nzioki

**Affiliations:** 1 Health Services Research Unit, KEMRI Wellcome Trust, Nairobi, Nairobi, 43640-00100, Kenya; 2 Environmental Health and Disease Control, Jomo Kenyatta University of Agriculture and Technology, Nairobi, Nairobi, 62 000 – 00200, Kenya; 3 Directorate of Veterinary Services, Ministry of Agriculture, Livestock, Fisheries and Cooperatives, Nairobi, Nairobi, Kangemi 00605, Kenya

**Keywords:** Adherence, compliance, Iron and Folic Acid, postnatal, primigravida, multigravida, Kakamega, Kenya

## Abstract

**Background: **Maternal anaemia is a public health problem worldwide, and its aetiology is linked to iron deficiency. The high nutrient demand during pregnancy exacerbates the condition. To meet the increased nutritional demand, supplementation of iron and folic acid (IFA) is key. The supplements are provided freely to pregnant women during antenatal visits at public health facilities, however, their uptake and adherence in Kenya remain unacceptably low.

**Methods: **A hospital-based cross-sectional study involving 241 postnatal mothers seeking maternal and child healthcare (MCH) care at Kakamega level 5 hospital was conducted. Both quantitative and qualitative data were collected. Quantitative data were collected from 241 eligible postnatal mothers, while qualitative data were obtained through key informant interviews with community health volunteers and healthcare providers.

**Results: **There was a moderate adherence to IFA supplementation (60.6%) during pregnancy among postnatal mothers seeking MCH care at Kakamega level 5 hospital. Some of the reasons for non-adherence stated by the respondents included; IFA related side effects (41.3%), forgetfulness (37.3%) and bad smell of the IFA supplements (10.3%). Higher IFA adherence was noted among the primigravida participants (OR=2.704; 95% CI: 1.262, 5.793; p=0.010) compared to multigravida participants, and those with a higher knowledge level of anaemia (OR=3.215; 95% CI: 1.346, 7.68; p=0.009) compared to their counterparts with low anaemia knowledge. Other factors that showed correlation with IFA adherence were: IFA education, pregnancy counselling before conception and the number of antenatal care visits attained.

**Conclusion: **There is a moderate adherence to IFA supplementation during pregnancy among mothers seeking MCH at Kakamega level 5 hospital. The greatest impediments of IFA compliance during pregnancy are IFA side effects, forgetfulness and the bad smell of the IFA tablets. Therefore, providing IFA education to pregnant mothers incorporated with probable ways of managing the IFA side effects would contribute to IFA supplementation adherence.

## Introduction

Maternal anaemia remains a public health problem worldwide ^1^. The World Health Organization (WHO) defines anaemia among pregnant women as having haemoglobin levels of less than 11.0g/dl ^2^. Anaemia in pregnant women has been attributed to iron deficiency ^3^. The condition is exacerbated by the high nutrient demand during pregnancy ^4^. Globally, 38% of pregnant women have been reported to be anaemic with the highest burden being in Africa ^1^. The WHO reports that 55.8% of pregnant women in Sub Saharan Africa (SSA) are anaemic ^1^. In Kenya, it is estimated that 55.1% of pregnant women have iron-deficiency anaemia ^5^. A study conducted at Kakamega level 5 hospital noted a 38.9% anaemia prevalence among pregnant women seeking antenatal care (ANC) at the facility ^6^.

To reduce incidences of maternal anaemia, the WHO recommends iron and folic acid (IFA) supplementation to all pregnant women ^7^. According to WHO guidelines, daily 30mg–60mg of iron and 0.4 mg of folic acid supplements are essential to all pregnant women and their uptake should be commenced as early as possible once pregnancy has been confirmed ^8^. However, strict adherence to IFA is required for better outcomes ^9^. Evidence suggests that IFA uptake during pregnancy reduces the chances of iron-deficiency anaemia to a great extent ^10^. Moreover, adequate IFA ensures the wellness of the developing foetus by reducing incidences of neural tube defects, puerperal sepsis, preterm birth, congenital heart defects, and low birth weight ^7, 11^.

To ensure equitable access, the government provides IFA supplements to all pregnant women seeking ANC in public health facilities at no cost ^12^. However, despite all these efforts, adherence to IFA among pregnant women in Kenya remains poor ^12, 13^. Although there has been a gradual national upward trend in self-reported IFA adherence from 2.5% in 2010 ^14^ to 8% in 2014, it is clear that the adherence is still low. In western Kenya, where Kakamega level 5 hospital is located, IFA adherence is even lower compared to the national prevalence (6.9%) ^15^. 

The causes of poor adherence to IFA uptake in Kenya are multidimensional. However, inaccessibility to ANC services could be the leading contributor to this poor trend ^16^. Surprisingly, poor adherence has also been confirmed among pregnant women who regularly visit health facilities for ANC ^9, 17, 18^. Inadequate knowledge of anaemia, frequent stock-outs of IFA tablets in health facilities, and forgetfulness have been linked as key barriers to IFA supplementation adherence in various studies ^9, 19, 20^. The side effects resulting from IFA consumption also discourage pregnant mothers from IFA compliance ^13^. Although these side effects are common with IFA use, many pregnant women seem to be unaware that practices such as taking the supplements alongside meals or just before going to bed help to alleviate the side effects. The persistently high prevalence of maternal anaemia coupled with poor adherence to IFA supplementation creates a need for more understanding of the possible causes of the poor adherence trend. Therefore, this study sought to investigate the possible determinants of IFA supplementation adherence during pregnancy among postnatal mothers seeking maternal and child healthcare (MCH) care at Kakamega level 5 hospital.

## Methods

### Study site

The study was conducted at Kakamega level 5 hospital located in western Kenya. The hospital serves as the main referral public health facility in Kakamega county of Kenya. This facility is located within Kakamega town and has bed capacity of 449, according to the Kenya Master Health facility List (KMHFL) of 2021 ^21^.

### Study design

A cross-sectional study was conducted between May and August 2020. The study targeted postnatal mothers of 0–6 months post-delivery seeking MCH care at Kakamega level 5 hospital. The sample size was calculated based on Cochran formula ^22^ as shown below:


$$n=\frac{{\text{Z}}^{2}\text{P}(1-\text{P})}{{\text{d}}^{2}}$$

To achieve our desired sample size (n), we set the standard normal deviation (Z) at 1.96 with 5% as the level of accuracy (d). A prevalence (P) of 32.7% was used basing on the findings of a similar study conducted in a similar county of Kenya. A total of 339 participants were therefore needed (n) but since our target population of 0–6 months postnatal mothers was less than 10000, the sample size was adjusted as shown ^22^:


$$nf=\frac{\text{n}}{1+\text{n}/\text{N}}$$

The approximate total number of women that were anticipated to seek postnatal care (PNC) services during the 3 months of data collection in Kakamega level 5 hospital was attained by multiplying the average monthly PNC attendance by 3. According to the 2019 District Health Information system (DHIS2) data, the average number of women (with children aged 0–6 months) who sought MCH services at this facility every month was 277. We therefore anticipated that 831 mothers would be encountered during the 3 months of data collection (277 X 3=831). Therefore:


$$nf=\frac{339}{1+(339/831)}=241$$

We catered for nonresponse by proceeding to another respondent until the required sample size was achieved.

### Data collection

***Quantitative data – semi-structured questionnaire.*** Quantitative data were collected at the postnatal section of Kakamega level 5 hospital among postnatal mothers of 15–49 years. We targeted the postnatal mothers who had just delivered in the maternity wards and those who had brought their babies for a routine check-up within six months post-delivery. We were keen to ensure that all the postnatal mothers interviewed were seeking healthcare services related to MCH care. However, the postnatal mothers who were critically ill such that they wouldn’t speak to us comfortably were excluded from the study. An electronic version of a semi-structured questionnaire developed in the Research Electronic Data Capture tool (REDCap) was used to collect the data (see data dictionary codebook for questions in *Underlying data* ^23^). We scheduled appointments in two sessions every day; the morning session and the afternoon session. About 10 participants were interviewed each day with each of the interviews lasting for approximately 30 minutes. Study participants were recruited once they had obtained the MCH care that they needed.

The purpose of the study was explained to each potential participant after which a written consent was sought. Participants of less than 18 years were only interviewed if they were accompanied by a guardian who provided written consent on their behalf. Every potential study participant who consented to the study was directed to a separate room where a face-to-face interview was conducted. However, interviews with mothers who had just delivered were conducted at their respective bedsides in the ward.

The questionnaire used in the data collection was divided into five sections: socio-demographic characteristics of the participants, health system-related characteristics, participant’s knowledge of anaemia, participant’s attitude towards ANC healthcare provider and IFA adherence. Adherence to IFA supplementation was established by asking the participants about the average number of IFA tablets taken per week throughout the gestation period. Participant’s knowledge of anaemia was ascertained using a set of 10 questions focusing on anaemia causes, haemoglobin level boosting foods, consequences of anaemia and its prevention mechanisms. All the responses provided by the respondents were entered in REDCap ^22^, after which they were uploaded to KEMRI WELLCOME TRUST server every day.

***Qualitative data – key informant interviews.*** The quantitative data were complemented by the key informant interviews (KIIs) with healthcare providers and the community health volunteers (CHVs) attached to the hospital. The first author administered all the KIIs. A total of five healthcare providers purposively selected within the hospital and all the 11 CHVs attached to the hospital participated in the KIIs. Neither of the targeted healthcare providers nor CHVs refused to take part in the study. The health care providers who provided feedback to the KIIs comprised of: PNC in-charge, ANC in-charge, PNC nurse, PNC pharmacist-in-charge and the MCH nutritionist in-charge. The KIIs with the healthcare providers took place at the hospital within their respective departments during individuals’ shift. We were flexible in scheduling the KIIs with the healthcare providers and only spoke to each one of them at the time that seemed convenient for them. This was to ensure that the interviews did not compromise their service provision to the patients. The KIIs with CHVs either took place at their respective homes or within Kakamega level 5 hospital. Those who were interviewed at the facility had accompanied a patient to the facility to help them obtain care. We chose to interview some CHVs at their respective homes to avoid them incurring transport and other costs related to travelling to the facility. The KIIs reported in this study were based on different, but strongly related KII guides prepared to suit either CHVs or healthcare providers (see KII guide in the *Extended data* ^23^). All the KIIs lasted between 30–45 minutes and were audio-recorded after the participants had given written informed consent to participate. All the interviews were conducted in secluded areas to ensure privacy of the respondents.

### Data analysis

During analysis, the quantitative data were exported from the server to a Microsoft Excel CSV file after which a data cleaning process followed.

*Adherence to IFA supplementation:* This was the main outcome variable in this study. Respondents who took at least five IFA tablets per week throughout their gestation period were classified as adherent, while those who took less than five tablets per week throughout pregnancy were non-adherent.

*Knowledge of anaemia:* Anaemia knowledge was determined by scoring the responses to the ten questions assessing knowledge level of anaemia. The questions inquired on anaemia causes, consequences and its prevention. Each of the ten questions answered correctly was awarded a score of ‘1’ while a wrong response or admitting not being aware was awarded a score of ‘0’. The total expected score of anaemia knowledge was 10 and this was used to calculate the percentage score of each participant. The respondents with a score that was equal to or greater than 50% were assumed to have a higher knowledge of anaemia while those with a score below 50% were treated as having low knowledge of anaemia ^24^.

*Attitude towards ANC healthcare provider:* We also established the attitude of the respondents towards ANC healthcare provider using a Likert scale. Five questions were asked on attitude, with responses ranging from strongly disagree, disagree, neutral, agree and strongly agree. The responses were scored in ascending order, with strongly disagree being scored a ‘1’ while strongly agree was scored a ‘5’. The scores were summed up and later converted into percentages. Respondents were considered to have a positive attitude towards ANC healthcare provider if they scored at least 70%, while those who scored below 70% were assumed to have a negative attitude towards ANC healthcare provider ^25^.

The cleaned verified data were then imported to R statistical software version 3.5.2 [2018–12–20] for analyses. The respondents’ socio-demographic characteristics and the prevalence of IFA compliance were summarized using descriptive statistics such as means, proportions and percentage frequencies. Some variables were also re-grouped into categories based on certain set thresholds. For example, mothers’ age was re-categorized into groups, IFA adherence classified as adherent or non-adherent, gravidity as primigravida and multigravida, while the education level of the mother was categorized into primary, secondary and college levels. Equally, the number of ANC visits attained by the mothers were segregated into two and below ANC visits and at least 3 ANC visits. Associations between adherence to IFA supplementation and its putative determinants were investigated through univariable and multivariable logistic regression. P values of less than 0.05 were interpreted as significant.

Qualitative data analysis began with the transcription of the recorded audio, but we did not return the transcripts to the interviewees for checking and verification. The first and second author conducted a deductive coding to organize the data into themes using the qualitative data analysis software NVivo 12 (QSR International, Australia).

### Ethical clearance

Ethical clearance for this study was sought from the Ethical Review Board of Daystar University (approval number: DU-ERB- 000415). Daystar University Ethical Review Board was considered for ethical approval as we were keen to have the study protocol reviewed within the anticipated timelines. The National Commission for Science, Technology and Innovation (NACOSTI) granted the research license. Approvals were also obtained from the hospital in-charge of Kakamega level 5 hospital and the head of the PNC section.

## Results

### Socio-demographic characteristics of the respondents

A total of 241 mothers seeking MCH care at Kakamega level 5 hospital were interviewed between May and August 2020. The average age of the mothers was 24.9±5.3 years with an age range of 15–43 years. As shown in Table 1, the majority (68.1%) of the respondents belonged in the age category of 19–29 years. Only a few of the respondents had formal employment (19.9%) while 22.4% were students. Most of the mothers interviewed (41.1%) had attained a secondary school level of education. Also, all the respondents admitted to belonging to one religion or another with the majority being Christians (95.9%). More than three-quarters of the mothers (85.5%) resided at a distance greater than 30 minutes to their ANC facilities with motorcycles being the most preferred means of transport (62.2%). A large proportion of the mothers had a high knowledge of anaemia (85.5%). All the respondents admitted to having been given IFA at any one point during ANC and out of these, slightly over half (51.0%) reported having experienced side effects as a result of IFA consumption.

**Table 1.** Socio-demographic characteristics of the respondents, Kakamega level 5 hospital, Kenya, 2020.

Variable	n	%	95% CI
**Mother’s age (years)**			
≤18	26	10.8	7.3, 15.6
19–29	164	68.1	61.7, 73.9
≥30	51	21.2	16.3, 27.0
**Occupation**			
Formal	48	19.9	15.2, 25.6
Non-formal	70	29.0	23.5, 35.3
Housewife	69	28.6	23.1, 34.9
Student	54	22.4	17.4, 28.3
**Education level**			
Primary	48	19.9	15.2, 25.6
Secondary	99	41.1	34.9, 47.6
College	94	39.0	32.9, 45.5
**Marital status**			
Married	172	71.4	65.1, 76.9
Single	69	28.6	23.1, 34.9
**Religion**			
Christian	231	95.9	92.3, 97.9
Muslim	10	4.1	2.1, 7.7
**Distance to ANC facility**			
>30 Minutes	206	85.5	80.2, 89.5
≤30 Minutes	35	14.5	10.5, 19.8
**Transport mode to ANC facility**			
Walking	73	30.3	24.6, 36.6
Motorcycle	150	62.2	55.8, 68.3
Matatu	18	7.5	4.6, 11.7
**Anaemia knowledge**			
Low	35	14.5	10.5, 19.8
High	206	85.5	80.2, 89.5
**IFA side effects**			
No	118	49.0	42.5, 55.4
Yes	123	51.0	44.6, 57.5

### Obstetric related characteristics of the respondents

As shown in Table 2, out of all the respondents interviewed, over two-thirds (73.9%) had completed at most 3 months post-delivery. Almost half of the mothers were multigravida (47.7%) and a few reported having had either a birth complication (12.1%) or miscarriage (4.6%). Most of the respondents (76.3%) had never had a pregnancy counselling session with a healthcare provider before getting pregnant. The majority of the mothers (71.8%) sought their first ANC services with a gestation period of more than 8 weeks and many of them attended ANC at least 3 times (92.9%). 

**Table 2.** Obstetric related characteristics of the studied respondents, Kakamega level 5 hospital, Kenya, 2020.

Variables	n	%	95% CI
**Postpartum duration**			
0–3 Months	178	73.9	67.8, 79.2
4–6 Months	63	26.1	20.8, 32.3
**Gravidity**			
Primigravida	126	52.3	45.8, 58.7
Multigravida	115	47.7	41.3, 54.2
**Birth complication**			
No	212	88.0	83.0, 91.7
Yes	29	12.1	8.3, 17.0
**Miscarriage**			
No	230	95.4	91.8, 97.6
Yes	11	4.6	2.4, 8.2
**Counselling before pregnancy**			
No	184	76.3	70.4, 81.5
Yes	57	23.7	18.5, 29.6
**First ANC gestation**			
≤8 weeks	68	28.2	22.7, 34.4
>8 weeks	173	71.8	65.6, 77.3
**ANC visits**			
≥3 Times	224	92.9	88.7, 95.7
≤2 Times	17	7.1	4.3, 11.3

**Figure 1.**
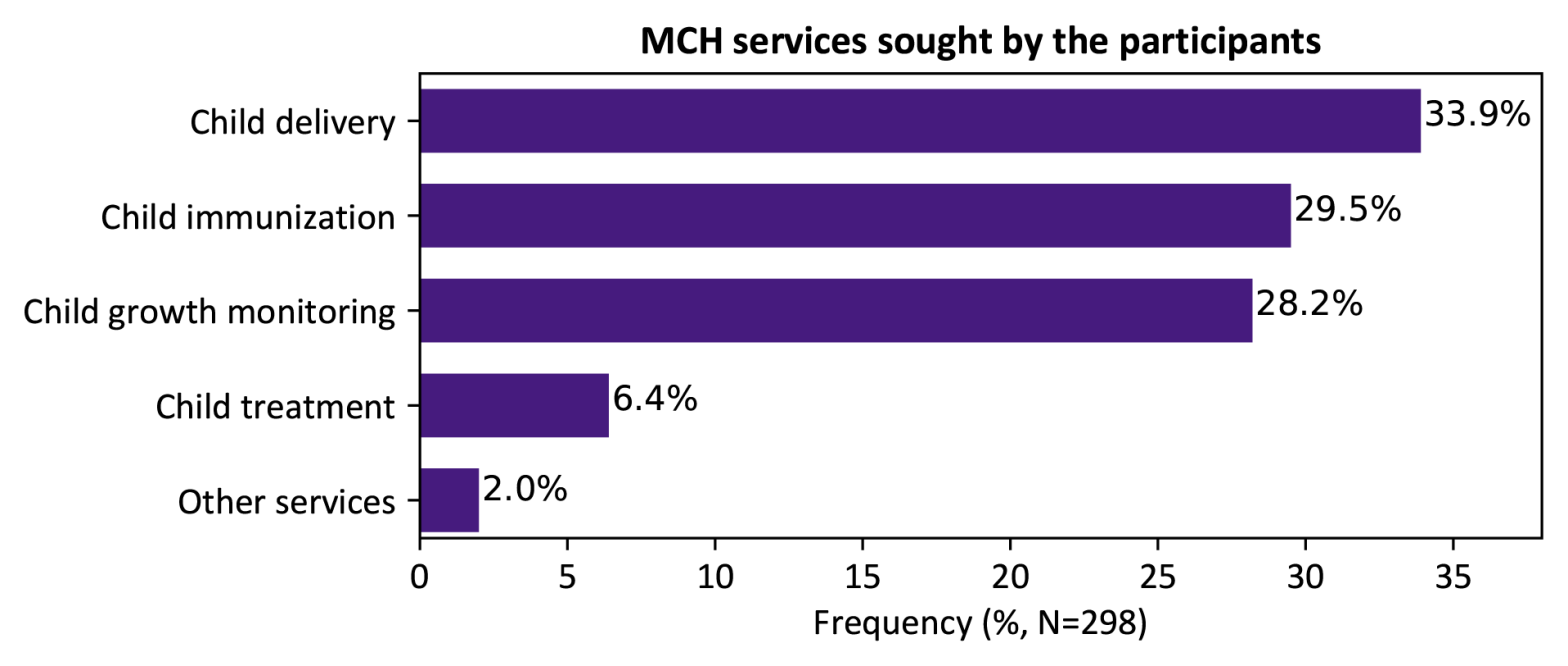
Maternal and child healthcare services sought by respondents, Kakamega level 5 hospital, Kenya, 2020.

Although most of the mothers interviewed in this study had visited the facility to obtain delivery care (33.9%), other MCH services sought included; child immunization (29.5%), child growth monitoring (28.2%), child treatment (6.4%) among others, as shown in Figure 1.

### Prevalence of IFA adherence

The overall prevalence of IFA adherence among the studied respondents was 60.6% with 146 (146/241) mothers having reported taking at least five IFA tablets per week throughout pregnancy. Out of the IFA non-compliant cohort, slightly more than one third (35.3%) consumed three to four IFA tablets per week on average throughout pregnancy. The highest adherence was observed among the single mothers (79.7%), followed by younger mothers of 18 years and below (76.9%). There was a similar trend of IFA compliance among Muslims and Christians, 60% and 60.6%, respectively. About eight in every 10 of the mothers who never experienced any IFA related side effects (75.9%) were IFA compliant. Less than half (41.7%) of the respondents with a primary education level took IFA as recommended. Mothers who resided within 30 minutes distance from their ANC facilities had a slightly higher adherence (61.2%) as compared to their counterparts residing more than 30 minutes away from their facilities of ANC (57.1%), as shown in Table 3.

**Table 3.** Prevalence of IFA adherence among the respondents, Kakamega level 5 hospital, Kenya, 2020.

		IFA adherence					
Variables		Average number of IFA tablets taken by respondents per week					
	Total (N)	0-2	%	3-4	%	5-7	%
**Mother’s age**							
≤18	26	0	0	6	23.1	20	76.9
19-29	164	6	3.7	56	34.1	102	62.2
≥30	51	4	7.8	23	45.1	24	47.1
**Education level**							
Primary	48	1	2.2	27	56.3	20	41.7
Secondary	99	4	4.0	29	29.3	66	66.7
College	94	5	5.3	29	30.9	60	63.8
**Marital status**							
Married	172	8	4.7	73	42.4	91	52.9
Single	69	2	2.9	12	17.4	55	79.7
**Distance to ANC**							
>30 mins	35	3	8.6	12	34.3	20	57.1
≤30 mins	206	7	3.4	73	35.4	126	61.2
**Side effects**							
No	118	4	3.7	32	29.6	82	75.9
Yes	123	6	5.0	53	43.8	64	52.9
**Occupation**							
Formal	48	3	6.3	14	29.2	31	64.6
Non-formal	70	2	2.9	30	42.9	38	54.3
Housewife	69	3	4.3	27	39.1	39	56.5
Student	54	2	3.7	14	25.9	38	70.4
**Religion**							
Christian	231	10	4.3	81	35.1	140	60.6
Muslim	10	0	0	4	40.0	6	60.0
** Total**		**10**	**4.149**	**85**	**35.3**	**146**	**60.6**

Overall Iron and Folic Acid (IFA) prevalence **(60.6%)**.

A total of 95 (39.4%) respondents were not IFA compliant. Out of this cohort, the majority of the mothers (41.3%) attributed the non-compliance to IFA related side effects. Other common reasons for non-adherence stated by the respondents were: forgetfulness (37.3%), bad smell of the IFA tablets (10.3%) and pharmacophobia (6.3%), as shown in Table 4.

**Table 4.** Reasons for non-compliance as stated by the respondents, Kakamega level 5 hospital, Kenya, 2020.

Non-adherence reason	Responses	%	95% CI
Side effects	52	41.3	32.68, 50.40
Forgetfulness	47	37.3	29.00, 46.41
Bad smell	13	10.3	5.83, 17.32
Pharmacophobia	8	6.3	2.98, 12.53
Make the fetus grow bigger	4	3.2	1.02, 8.42
Unaware of IFA importance	1	0.8	0.04, 4.99
Ran out of supplements	1	0.8	0.04, 4.99
**Total**	**126**	**100**	

**Figure 2.**
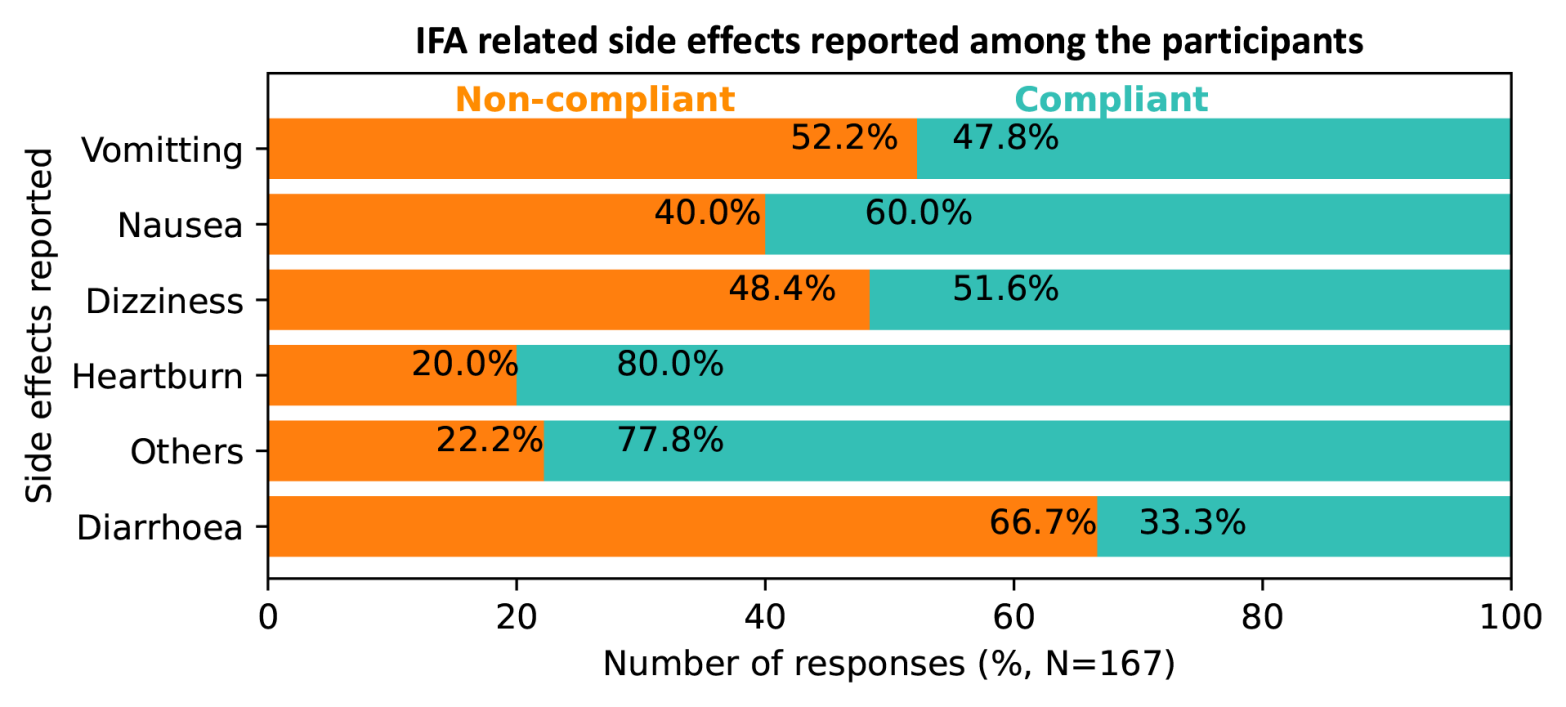
Variations in iron and folic acid (IFA) compliance according to the side effects experienced among respondents, Kakamega level 5 hospital, Kenya, 2020.

A total of 123 (51%) mothers experienced IFA related side effects. Out of this, almost half (48.0%) were non-compliant. The most prevalent side effects were vomiting (41.3%), nausea (26.9%) and dizziness (18.6%). Slightly over half (52.2%) of the mothers who experienced vomiting while on IFA were non-compliant. About 48.4% of those who felt dizzy due to IFA consumption and the majority of the mothers who experienced IFA related diarrhoea could not comply with IFA supplementation ( Figure 2).

### Socio-demographic determinants of IFA adherence

Investigation of IFA adherence trends through univariable logistic regression was conducted. Specifically, it was noted that postnatal mothers who were at least 30 years of age were less likely to be IFA compliant as compared to their younger counterparts of 18 years and below, (OR=0.267, 95% CI: 0.086, 0.741; p=0.015). Mothers with a primary level of education had their odds of being IFA compliant reduced by 60% as compared to mothers who had acquired a college level of education, (OR=0.405, 95% CI: 0.196, 0.819; p=0.013). Similarly, the study established reduced odds of IFA adherence among the respondents who had attended ANC for at most two times as well as those who had reported experiencing IFA related side effects, (OR=0.327, 95%CI: 0.109, 0.893; p=0.034) and (OR=0.476, 95% CI: 0.279, 0.804; p=0.006), respectively. Unlike mothers in marriage, single mothers were 3.5 times more likely to be IFA compliant (OR=3.497,95% CI: 1.852, 6.971; p<0.001). The primigravida mothers were almost four-fold more likely to be IFA compliant as compared to multigravida mothers (OR=3.559, 95%CI: 2.081, 6.187; p<0.001). Equally, the respondents who seemed to have higher anaemia knowledge were noted to have elevated odds of adhering to IFA supplementation, (OR=2.676, 95% CI: 1.296, 5.680; p=0.009) ( Table 5).

**Table 5.** Univariable logistic regression model demonstrating socio-demographic risk factors for IFA adherence, Kakamega level 5 hospital, Kenya, 2020.

Variables	Beta	Std. Error	Z value	OR (95% CI)	P value
**Mother’s age (Years)**					
18	Ref				
19–29	-0.706	0.493	-1.434	0.494 (0.173, 1.23)	0.152
≥30	-1.322	0.544	-2.432	**0.267 (0.086, 0.741)**	**0.015**
**Education level**					
College	Ref				
Secondary	0.125	0.303	0.414	1.133 (0.626, 2.055)	0.679
Primary	-0.905	0.363	-2.491	**0.405 (0.196, 0.819)**	**0.013**
**Marital status**					
Married	Ref				
Single	1.252	0.t336	3.725	**3.497 (1.852, 6.971)**	**<0.001**
**Gravidity**					
Multigravida	Ref				
Primigravida	1.269	0.278	4.575	**3.559 (2.081, 6.187)**	**<0.001**
**Birth complication**					
No	Ref				
Yes	-0.412	0.398	-1.036	0.662 (0.303, 1.457)	0.300
**Anaemia knowledge**					
Low	Ref				
High	0.984	0.374	2.629	**2.676 (1.296, 5.680)**	**0.009**
**ANC visits**					
≥3	Ref				
≤2	-1.117	0.526	-2.124	**0.327 (0.109, 0.893)**	**0.034**
**Distance to ANC**					
>30 mins	Ref				
≤30 mins	0.167	0.370	0.450	1.181 (0.564, 2.432)	0.653
**IFA side effects**					
No	Ref				
Yes	-0.742	0.269	-2.754	**0.476 (0.279, 0.804)**	**0.006**

Standard Error (Std. Error), odds Ratio (OR), Significant (P<0.05).

In order to understand the independent association between IFA adherence and the socio-demographic factors, all the variables with crude P values of less than 0.2 were fitted to a multivariable model. These included: mother’s age, mother’s education level, marital status, gravidity, mother’s knowledge of anaemia, number of ANC visits, and IFA related side effects. The multivariable model confirmed the independent correlation between IFA adherence and gravidity (OR=2.704, 95% CI: 1.262, 5.793; p=0.010), mother’s anaemia knowledge (OR=3.215, 95% CI: 1.346, 7.68; p=0.009), number of ANC visits (OR=0.273, 95% CI: 0.081, 0.916; p=0.036) and IFA side effects (OR=0.444, 95% CI: 0.246, 0.803; p=0.007), as shown in Table 6.

**Table 6.** Multivariable logistic regression model demonstrating socio-demographic risk factors for IFA adherence, Kakamega level 5 hospital, Kenya, 2020.

Variables	Estimate	Std. Error	Z value	OR (95% CI)	P value
**Mother’s age (Years)**					
≤18	Ref				
19-29	-0.356	0.615	-1.578	0.701 (0.21, 2.339)	0.563
≥30	-0.196	0.747	-0.263	0.822 (0.19, 3.553)	0.793
**Education level**					
College	Ref				
Secondary	0.295	0.359	0.822	1.344 (0.664, 2.718)	0.411
Primary	-0.481	0.428	-1.125	0.618 (0.267, 1.429)	0.260
**Marital status**					
Married	Ref				
Single	0.712	0.447	1.592	2.037 (0.848, 4.893)	0.111
**Gravidity**					
Multigravida	Ref				
Primigravida	0.995	0.389	2.559	**2.704 (1.262, 5.793)**	**0.010**
**Anaemia knowledge**					
Low	Ref				
High	1.168	0.444	2.629	**3.215 (1.346, 7.68)**	**0.009**
**ANC visits**					
≥3	Ref				
≤2	-1.300	0.618	-2.102	**0.273 (0.081, 0.916)**	**0.036**
**IFA side effects**					
No	Ref				
Yes	-0.811	0.302	-2.687	**0.444 (0.246, 0.803)**	**0.007**

Standard Error (Std. Error), odds Ratio (OR), Significant (P<0.05)

### Health system-related determinants of IFA adherence

Univariable analysis was used to identify possible health system related factors influencing IFA supplementation adherence. Mothers who received education on IFA were almost three times more likely to be IFA compliant as compared to those who did not receive education on IFA (OR=2.728, 95% CI: 1.297, 5.921; p=0.009). Similarly, attending pregnancy counselling sessions prior to conception played a significant role on IFA adherence. The study revealed that mothers who sought pregnancy counselling sessions were more than twice likely to be IFA compliant as compared to their counterparts who never attended pregnancy counselling sessions before conception (OR=2.415, 95% CI: 1.262, 4.859; p<0.01). Unlike mothers who had a negative attitude towards ANC healthcare provider, mothers who had a positive attitude towards ANC healthcare provider were up to 2.4 times more likely to be IFA compliant (OR=2.357, 95% CI: 1.124, 5.052; p=0.024), as shown in Table 7.

**Table 7.** Univariable logistic regression model demonstrating health system-related risk factors for IFA adherence, Kakamega level 5 hospital, Kenya, 2020.

Variables	Beta	Std. Error	Z value	OR (95% CI)	P value
**IFA education**					
No	Ref				
Yes	1.004	0.384	2.611	**2.728 (1.297, 5.921)**	**0.009**
**Pregnancy counselling**					
No	Ref				
Yes	0.882	0.342	2.581	**2.415 (1.262, 4.859)**	**<0.01**
**Turnaround Time**					
Longer than expected	Ref				
As expected,	0.342	0.321	1.067	1.408 (0.747, 2.640)	0.286
**IFA stock out**					
No	Ref				
Yes	-0.253	0.400	-0.634	0.776 (0.355, 1.723)	0.526
**Attitude towards ANC healthcare provider**					
Negative	Ref				
Positive	0.856	0.381	2.253	**2.357 (1.124, 5.052)**	**0.024**

According to the KIIs with CHVs, poor attitude towards ANC healthcare provider is partially contributed by the long turnaround time taken during ANC visits.

“ *There are still others who prefer enrolling for ANC a bit late say three to four months due in pregnancy. They just want to buy time, especially when they think of the long queue that awaits them at the ANC. They complain that they take a lot of time at ANC*…” (KII: CHV).

All the health system related factors that showed a p≤0.2 in the univariable logistic regression were subjected to a multivariable model. Precisely, IFA education, having had a pregnancy counselling session and mother’s attitude towards ANC healthcare provider were adjusted for in the multivariable model. Having had a pregnancy counselling session with a healthcare provider before conception and having received education on IFA showed independent association with IFA adherence, (OR=2.086, 95% CI: 1.071, 4.255; p=0.036) and (OR=2.372, 95% CI: 1.109, 5.218; p=0.028) respectively ( Table 8).

**Table 8.** Multivariable logistic regression model demonstrating health system-related risk factors for IFA adherence, Kakamega level 5 hospital, Kenya, 2020.

Variables	Beta	Std. Error	Z value	AOR (95% CI)	P value
**IFA education**					
No	Ref				
Yes	0.864	0.392	2.203	**2.372 (1.109, 5.218)**	**0.028**
**Pregnancy counselling**					
No	Ref				
Yes	0.735	0.350	2.102	**2.086 (1.071, 4.255)**	**0.036**
**Attitude towards ANC healthcare provider**					
Negative	Ref				
Positive	0.641	0.400	1.637	1.900 (0.884, 4.150)	0.102

The correlation between hospital IFA stockouts and IFA adherence was not statistically significant. However, KIIs with healthcare workers revealed that the facility runs out of the supplements at times, although not very often. Despite the existence of clear channels on how to source for the IFA tablets, it would take a lot of time for the officer in charge to be notified of the IFA stock-outs. The respondent felt that the situation was exacerbated by delayed communications among the staff and this, to some extent, resulted to missed opportunities for taking IFA.

“… *As I said, we need to have a nutritionist in every room but that’s almost impossible for now. So sometimes when I’m compiling monthly reports, I notice that the mothers have not been issued with the supplements for some time. When you make follow-ups, you will be told that IFA had run out of stock. But the problem is that they did not communicate because I could have looked for the supplements somewhere else even if it means borrowing from another facility…”* (KII: healthcare provider).

We also learned that the ANC healthcare providers experienced challenges in providing IFA education and general MCH talks to ANC seeking mothers. They attributed this to health system disruptions caused by the COVID-19 pandemic whereby the ANC services were shifted to another department to create more space for COVID 19 patients as indicated in transcripts below.

“ *Before we came here, we got some space at the eye clinic. But it was even smaller than this. We tried giving health talks there and you would find yourself talking to a group of eye patients, ANC mothers and under-five mothers. It was such a bad confusion and we had to move from there to this orthopaedic department. The space here still doesn’t allow for such talks though. That is as it is for now…”* (KII: healthcare provider).
*“Did you notice that even the sitting space is not enough? We have no space for more benches. Some mothers are standing as you can see. In fact, this has impacted negatively on our ANC visiting trends. It seems mothers are no longer motivated to come for ANC as they feel that the place is congested to an extend that they miss a place to sit…”* (KII: healthcare provider).

As indicated in the following verbatim, healthcare workers at the ANC section experience a heavy workload very often. This is partly due to the limited number of staff as well as the rigorous writing involved in their line of duty.

*“I’m the only one here and the two ladies you see over there are interns, the other lady is a casual on the hospital’s payroll. And there is a lot of writing involved by the way. We do not have enough computers and the few that we have keep on hanging…”* (KII: healthcare provider).

## Discussion

There is moderate adherence to IFA supplementation (60.6%) during pregnancy among mothers seeking MCH care at Kakamega level 5 hospital. The finding is consistent with similar studies conducted in a sub-city of Ethiopia (60.9%), as well as a metropolitan area of Ghana (58.8%) ^26, 27^. The IFA supplementation prevalence was however higher as compared to 32.7% reported in Kiambu county of Kenya ^13^ and 20.3% among rural communities of North Western Tanzania ^28^, but lower than 71% reported in India ^29^ and 68.6% in Niger ^30^. The moderately higher IFA supplementation prevalence recorded in the current study could be attributed to the fact that the study was conducted in a hospital located within a town. This implies that the hospital is very accessible and the mothers seeking MCH care within the hospital have more access to information as they are likely to be residing closer to town. Nevertheless, the moderate IFA adherence at Kakamega level 5 hospital suggests that pregnant mothers seeking MCH care in this facility and their unborn babies are still exposed to some risks of gestational maternal anaemia. This is because pregnant mothers are expected to comply with daily IFA uptake throughout the gestation period as in accordance with the WHO guidelines ^8^.

Participants who experienced IFA related side effects were less likely to be IFA compliant. Equally, most of the respondents who admitted not having adhered to IFA supplementation during their gestation period mentioned side effects (41.3%), forgetfulness (37.3%) and the bad smell of the supplements (10.3%) as the main challenges. Similar reasons for non-adherence have also been reported among pregnant women in India ^31^ and Ghana ^27^. In Ethiopia, Nasir *et al.* observed that more than half of the mothers who were IFA non-compliant either attributed the poor trend to IFA related side effects or forgetfulness ^32^. Most mothers do not seem to know how to manage IFA related side-effects and this makes them give up on the supplements quite easily. Out of the 123 mothers who reported to have experienced some side effects due to IFA consumption, almost 90% of them either stopped taking the supplements or let the side effects subside on their own. There is a need for health care providers to include education on the management of IFA related side effects as part of ANC counselling. Practices such as taking IFA alongside meals or just before going to bed as well as eating plenty of vegetables and fruits have been linked to reduced IFA side effects ^13^. Forgetfulness among IFA users could be reduced by encouraging mothers to embrace modern technology reminders or take the supplements at specific times, such as after meals, every morning, and just before going to bed. The women could also ask a reliable member of the family or friend to remind them to take the supplements.

Gravidity is a great determinant of IFA adherence. Primigravida mothers were almost three times more likely to be IFA compliant as compared to multigravida respondents. This is in line with other studies conducted in Kenya and India ^13, 31^. However, this finding is not consistent with Alemayu *et al.* and Niguse *et al.* who observed higher odds of IFA compliance among multigravida mothers in Ethiopia, ^17, 33^. The low IFA compliance among the multigravida mothers could be attributed to their experience with delivery and IFA usage. Some studies have shown that women who experience unpleasant side effects with IFA or have a history of good birth outcomes may not appreciate the need to adhere to IFA in their consecutive pregnancies ^34^. Therefore, sensitizing multigravida mothers on the importance of adhering to IFA supplementation during subsequent pregnancies regardless of the previous past experiences is key.

Mothers who attained fewer ANC visits were exposed to IFA non-adherence. Precisely, the respondents who attained a maximum of two ANC visits had up to 70% reduced chances of being IFA compliant. A large population-based study conducted among pregnant women in SSA noted higher odds of IFA compliance among women with at least four ANC visits ^35^. In Ethiopia, Molla *et al.* observed that women with at least four ANC visits were almost seven times more likely to be IFA compliant while Tarekegn *et al.* reported higher IFA compliance among women who had attained at least 3 ANC visits, ^36, 37^. Generally, ANC visits correlate positively with IFA adherence. A higher number of ANC visits indicates more frequency of contact between the mother and the healthcare provider. This provides a good opportunity for the healthcare provider to encourage the mothers to use the supplements as required. The mothers also get their supplements replenished through such visits to ensure that they do not run out of IFA. Besides, they could share any challenges encountered while taking the supplements with the healthcare provider and be advised accordingly. The WHO recommends at least eight ^3^ ANC visits in pregnancy and therefore encouraging pregnant women to complete the recommended number of visits is likely to have a positive influence on IFA compliance.

Women who have a higher knowledge of anaemia were more than three times likely to be IFA adherent as compared to their counterparts of low anaemia knowledge. Similar findings have also been reported in other studies elsewhere, ^27, 36, 38^. It is possible that having higher knowledge of anaemia enables a mother to understand the aetiology of anaemia, its prevention measures as well as the deleterious effects that the condition could cause to the mother and her unborn baby. This makes the mothers appreciate the importance of taking IFA as recommended.

Pregnancy counselling before conception and ANC education on the importance of IFA adherence are good ways of ensuring compliance with the supplements. This study revealed that women who received education on the importance of IFA during ANC were 2.4 times more likely to be IFA compliant as compared to their counterparts who had no education with regards to IFA supplementation. Just like IFA education, mothers who attended pregnancy counselling sessions before conception had higher odds of IFA compliance. This is consistent with other reports where IFA education during ANC or pregnancy counselling improved the uptake of the supplements ^13, 26^. Being educated on the importance of IFA helps mothers to appreciate the need to take the supplements consequently leading to compliance. Unfortunately, KIIs with ANC health care workers revealed that the staff were almost unable to provide this crucial education to ANC mothers as at the time of the study due to health system disruptions caused by the COVID-19 pandemic ^39^ that led to a shift in location of ANC services. Moreover, the understaffing at the ANC department and the rigorous documentation involved within this section barely leaves enough opportunity for individual health talk between a mother and the health care provider. The Ministry of Health should put measures in place to ensure uninterrupted continued provision of essential health care services such as MCH even in times of pandemics like COVID-19.

### Study limitation

The study was likely to have been affected by recall bias as the mothers had to recall their IFA usage trends during their gestation period. However, we minimized this bias by only selecting mothers of 0–6 months post-delivery.

## Conclusion

There is moderate adherence to IFA supplementation during pregnancy among mothers seeking MCH at Kakamega level 5 hospital. This suggests that there is still a need for improvement to meet the WHO recommendations where pregnant mothers are encouraged to take IFA supplements every day throughout pregnancy. The greatest impediments of IFA compliance are IFA related side effects, forgetfulness and the bad smell of the IFA tablets. Therefore, education on how to cope up with such anticipated outcomes should be provided to all pregnant mothers during ANC visits.

## Data availability

### Underlying data

Harvard Dataverse: Replication Data for: Adherence to Iron and Folic Acid supplementation during pregnancy among postnatal mothers seeking Maternal and Child Healthcare at Kakamega level 5 hospital in Kenya, https://doi.org/10.7910/DVN/ZEYYSZ ^23^.

The interview transcripts derived from the KIIs conducted in this study have not been made available for open access because this would violate the privacy that we initially assured all our study participants. Intermediary data in the form of quotes are avaliable throughout the Results section that reflect the data collected. Reasonable access to the interview transcripts may be granted after the application of access permission by downloading and filling this online form and then sending it to the Data Governing Committee at KEMRI-Wellcome Trust ( dgc@kemri-wellcome.org). The applicant must however prove beyond reasonable doubt that the confidentiality of the study participants will not be compromised in any way before data access is granted.

### Extended data

Harvard Dataverse: Replication Data for: Adherence to Iron and Folic Acid supplementation during pregnancy among postnatal mothers seeking Maternal and Child Healthcare at Kakamega level 5 hospital in Kenya, https://doi.org/10.7910/DVN/ZEYYSZ ^23^.

This project contains the following extended data:

- KII guide (CHV)
- KII guide (healthcare provider)

### Reporting guidelines

Harvard Dataverse: COREQ checklist for ‘Adherence to Iron and Folic Acid supplementation during pregnancy among postnatal mothers seeking Maternal and Child Healthcare at Kakamega level 5 hospital in Kenya’, https://doi.org/10.7910/DVN/ZEYYSZ ^23^.

Data are available under the terms of the Creative Commons Attribution 4.0 International license (CC-BY 4.0).
