# Supplementary material for: Adherence to iron and folic acid supplementation during pregnancy among postnatal mothers seeking maternal and child healthcare at Kakamega level 5 hospital in Kenya: a cross-sectional study
Source: Wellcome Open Res. 2021 Jul 5;6:80. Originally published 2021 Apr 12. [Version 2] doi: 10.12688/wellcomeopenres.16699.2 (PMC8366300; doi:10.12688/wellcomeopenres.16699.2)
Supplement: Peer review history

# Peer review: Reviewer response for version 2

**DOI:** 10.21956/wellcomeopenres.18803.r44785

**Published:** 2021-08-13

**Authors:** Katherine L. Dickin

**Affiliations:** 1 Master of Public Health Program, Population Medicine and Diagnostic Sciences, Cornell University, Ithaca, NY, USA

The authors have responded fully to my comments and made appropriate revisions to the article. I have no further comments.

Is the work clearly and accurately presented and does it cite the current literature?

Yes

If applicable, is the statistical analysis and its interpretation appropriate?

Partly

Are all the source data underlying the results available to ensure full reproducibility?

No source data required

Is the study design appropriate and is the work technically sound?

Yes

Are the conclusions drawn adequately supported by the results?

Partly

Are sufficient details of methods and analysis provided to allow replication by others?

Partly

Reviewer Expertise:

Maternal and child nutrition, social and behavior change, qualitative and mixed methods research, nutrition program implementation and effectiveness

I confirm that I have read this submission and believe that I have an appropriate level of expertise to confirm that it is of an acceptable scientific standard.

# Peer review: Reviewer response for version 2

**DOI:** 10.21956/wellcomeopenres.18803.r44786

**Published:** 2021-07-20

**Authors:** Martin N. Mwangi

**Affiliations:** 1 Training and Research Unit of Excellence (TRUE), College of Medicine, University of Malawi, Blantyre, Malawi

I have reviewed the edited paper and noted that the requested edits were addressed fully. I have no further comments to make.

Is the work clearly and accurately presented and does it cite the current literature?

Yes

If applicable, is the statistical analysis and its interpretation appropriate?

Partly

Are all the source data underlying the results available to ensure full reproducibility?

No source data required

Is the study design appropriate and is the work technically sound?

Yes

Are the conclusions drawn adequately supported by the results?

Yes

Are sufficient details of methods and analysis provided to allow replication by others?

Partly

Reviewer Expertise:

Anaemia and Iron Deficiency Anaemia in pregnancy and early childhood, Nutrition and Infectious Diseases, Nutritional epidemiology, Public Health Nutrition.

I confirm that I have read this submission and believe that I have an appropriate level of expertise to confirm that it is of an acceptable scientific standard.

# Peer review: Reviewer response for version 1

**DOI:** 10.21956/wellcomeopenres.18414.r43854

**Published:** 2021-06-15

**Authors:** Martin N. Mwangi

**Affiliations:** 1 Training and Research Unit of Excellence (TRUE), College of Medicine, University of Malawi, Blantyre, Malawi

This is a well written paper about a study that sought to investigate the possible determinants of Iron and Folic Acid (IFA) supplementation adherence during pregnancy among postnatal mothers seeking maternal and child healthcare at a referral hospital in Western Kenya. Using a mixed methods approach, the authors explore barriers to IFA uptake among pregnant women.

**Introduction**

 The background information section is well written. Important and recent references on the topic are cited. In paragraph 2, as part of our research in the same Western region of Kenya, the effect of iron supplementation on low birth weight was clearly demonstrated and thus this work deserves mention in relation to the effect of iron supplements on birth weight (see cited article).

**Methods**

 The methods section is well structured and contains most of the relevant information.

1. Adherence to IFA supplementation was the primary outcome and was defined as taking at least five IFA tablets per week throughout the gestation period. The authors do not explain why other less strict definitions such as the WHO definition (iron folate supplements ≥90 days or 4 days per week during the pregnancy period) were not sufficient for this population. One wonders to what magnitude the conclusions would change if the criteria stated here (≥90 days or 4 days per week) is used.
2. Qualitative data – key informant interviews (KIIs): the KIIs with healthcare providers and the community health volunteers (CHVs) attached to the hospital were well received and accepted by the respondents. What is missing is an explanation of how the authors determined whether all vital information was already collected from a respondent. In addition, how the authors determined the stop point for the KIIs. Did they rely on a saturation point for example?

**Results**

 The results section is clearly structured with adequate tables and figures. Because correlations are not causal the authors should be careful not to ascribe causality to otherwise correlated outcomes.

**Discussion**

 Paragraph one of the discussion reports the main finding of the study. More comparative data on the same outcome from other Kenyan counties could have been reported to aid generalisability. 

 Paragraph two of the discussion ends with suggestions on how to reduce forgetfulness among IFA users. The authors should include recent developments or strategies such as the use of intravenous iron for example ferric carboxymaltose to combat maternal anaemia in pregnancy.

 Bad smell has been noted to be among the top three reasons for non-compliance as reported by the participants of the study. The discussion could explore ways to overcome bad smells, for example in the manufacture of iron gel capsules so that pregnant mothers do not smell or taste the capsules before ingesting them. Other strategies could be outlined as well.

 Recall bias is not adequately discussed in the study yet the primary outcome (adherence to IFA supplementation during pregnancy) relied on the ability of participants to recall past events. The recall period is long especially if a participant was recruited in the 3-6 months postpartum period. The paper could benefit from a discussion on strategies applied to reduce the impact of recall bias on the primary outcome.

**General edits:**

- Introduction, paragraph 3 first sentence should read: “To ensure equitable access, the government ***of Kenya*** provides…”
- Introduction, last sentence, the word “care” seems to be repeated.

Is the work clearly and accurately presented and does it cite the current literature?

Yes

If applicable, is the statistical analysis and its interpretation appropriate?

Partly

Are all the source data underlying the results available to ensure full reproducibility?

No source data required

Is the study design appropriate and is the work technically sound?

Yes

Are the conclusions drawn adequately supported by the results?

Yes

Are sufficient details of methods and analysis provided to allow replication by others?

Partly

Reviewer Expertise:

Anaemia and Iron Deficiency Anaemia in pregnancy and early childhood, Nutrition and Infectious Diseases, Nutritional epidemiology, Public Health Nutrition.

I confirm that I have read this submission and believe that I have an appropriate level of expertise to confirm that it is of an acceptable scientific standard, however I have significant reservations, as outlined above.

# Response

**Published:** 2021-06-29

**Authors:** Felix Bahati

**Affiliations:** KEMRI WELLCOME TRUST, Kenya

This is a well written paper about a study that sought to investigate the possible determinants of Iron and Folic Acid (IFA) supplementation adherence during pregnancy among postnatal mothers seeking maternal and child healthcare at a referral hospital in Western Kenya. Using a mixed methods approach, the authors explore barriers to IFA uptake among pregnant women.

**Introduction**

 The background information section is well written. Important and recent references on the topic are cited. In paragraph 2, as part of our research in the same Western region of Kenya, the effect of iron supplementation on low birth weight was clearly demonstrated and thus this work deserves mention in relation to the effect of iron supplements on birth weight (see cited article).

*We appreciate the reviewer for making us aware about this interesting study. The findings of the interventional study cited by the reviewer in Western Kenya proved that the supplements reduced the risk of low birth weight by 58% as well as prematurity. We have cited this work in paragraph 2 of our introduction to clearly show the significance of IFA supplementation.*

**Methods**

 The methods section is well structured and contains most of the relevant information.

1. Adherence to IFA supplementation was the primary outcome and was defined as taking at least five IFA tablets per week throughout the gestation period. The authors do not explain why other less strict definitions such as the WHO definition (iron folate supplements ≥90 days or 4 days per week during the pregnancy period) were not sufficient for this population. One wonders to what magnitude the conclusions would change if the criteria stated here (≥90 days or 4 days per week) is used.

*As the reviewer notes, t* *his study had slightly higher threshold of defining IFA adherence compared to other studies that used a lower threshold. Our adherence definition was motivated by the current WHO guidelines that require pregnant mothers to take IFA tablets daily throughout pregnancy. Unlike the previous guidelines, the current WHO guidelines are stressing on the need for daily intake rather than achieving the previous cut-off of 90 tablets. We reasoned that the daily IFA intake advocated by the WHO throughout gestation coupled with the current ANC guideline of 8 ANC visits from the previous 4, signifies the need for mothers to consume the supplements daily. We therefore designed our study with an aim of defining adherence that would be in line with the current guidelines which gives it a greater chance of having significant policy implication. We, however, acknowledge the reviewers thought that maybe there would be some significant changes were it that another less strict adherence was used. We have brought out this issue by explaining in our first paragraph of the discussion that probably this could be the reason as to why our adherence prevalence could not match other studies done elsewhere. Besides, we have added a second last paragraph in the discussion section which partly highlights this issue.*


* *

1. Qualitative data – key informant interviews (KIIs): the KIIs with healthcare providers and the community health volunteers (CHVs) attached to the hospital were well received and accepted by the respondents. What is missing is an explanation of how the authors determined whether all vital information was already collected from a respondent. In addition, how the authors determined the stop point for the KIIs. Did they rely on a saturation point for example?

*Actually, the KII guides used by the interviewer also served as the checklists to ensure that all the relevant information required was discussed during the interview. We conducted KIIs with two groups of respondents in this study; the CHVs and healthcare providers. We conducted KIIs with all the CHVs (11) attached to the hospital. This was because, we knew that most of them came from different sub-ethnicities within the area and therefore this would give us an opportunity to understand deeply the socio-cultural factors affecting IFA adherence (data not presented in this article). As for the healthcare providers, we were keen to cause very minimal inconveniences to care provision and therefore stopped immediately the themes reached the saturation point. We realize that this is a crucial information that we should have provided in the methodology section as the reviewer suggests. We added this information in the methodology section within the ‘Qualitative data – key informant interviews’ section.*

**Results**

 The results section is clearly structured with adequate tables and figures. Because correlations are not causal the authors should be careful not to ascribe causality to otherwise correlated outcomes.

*This is a very crucial observation that was also raised by reviewer #1. We have therefore rephrased all the sentences in the discussion section that we thought could be easily misinterpreted to mean causality when in real sense the scope of our analysis was correlation.*

**Discussion**

 Paragraph one of the discussion reports the main finding of the study. More comparative data on the same outcome from other Kenyan counties could have been reported to aid generalisability. 

*Although there is a paucity of data in this area, the authors have identified a few relevant studies conducted in other Kenyan counties and made the necessary comparison. Please see references …and…*

 Paragraph two of the discussion ends with suggestions on how to reduce forgetfulness among IFA users. The authors should include recent developments or strategies such as the use of intravenous iron for example ferric carboxymaltose to combat maternal anaemia in pregnancy.

*We commend the reviewer for this insight. We learnt that more than 1000mg doses of F* *erric carboxymaltose* *could be administered to mothers in smaller divided doses administered at an interval on 7 days. This could save the mothers from the daily intake of the IFA tablets. We have included this in paragraph two of our discussion as suggested by the reviewer.*

 Bad smell has been noted to be among the top three reasons for non-compliance as reported by the participants of the study. The discussion could explore ways to overcome bad smells, for example in the manufacture of iron gel capsules so that pregnant mothers do not smell or taste the capsules before ingesting them. Other strategies could be outlined as well.

*Although many participants in our study complained about the bad smell of these tablets, we had not thought of a way of eliminating the bad smell without interfering with the efficacy of the supplements. We think that the adoption of an iron gel capsule as suggested by the reviewer would be helpful in eliminating the smell. We have suggested this in our discussion as recommended by the reviewer. Unfortunately, we couldn’t think of other appropriate strategies that could help alleviate this problem but stand ready to cite them if the reviewer suggests such other strategies.*

 Recall bias is not adequately discussed in the study yet the primary outcome (adherence to IFA supplementation during pregnancy) relied on the ability of participants to recall past events. The recall period is long especially if a participant was recruited in the 3-6 months postpartum period. The paper could benefit from a discussion on strategies applied to reduce the impact of recall bias on the primary outcome.

*We have added a whole paragraph in the discussion section to address the recall bias issue. We have cited evidence supporting our recall period of 0-6 months. Generally, pregnancy is a very important event in the lifetime of most women. According to other researchers, when an event is salient, the respondents tend to remember it even 20 months afterwards. This coupled with overall assessment of an event i.e asking the respondent to gauge their overall use of IFA in gestation rather than specific weeks/stages of gestation makes it easier for participants to remember. We are therefore confident that the findings reported in this article are relevant and were not influenced by recall bias to a great extent. We have also edited table 2 and provided the total number of respondents that we interviewed at every monthly interval post-delivery as suggested by the reviewer. The limitation section has also been updated to clearly show the recall period was a weakness in this study.*

**General edits:**

- Introduction, paragraph 3 first sentence should read: “To ensure equitable access, the government ***of Kenya*** provides…”

*This has been rectified*

* *

- Introduction, last sentence, the word “care” seems to be repeated.

*This has been edited.*

- **Is the work clearly and accurately presented and does it cite the current literature?**

Yes

- **Is the study design appropriate and is the work technically sound?**

Yes

- **Are sufficient details of methods and analysis provided to allow replication by others?**

Partly

- **If applicable, is the statistical analysis and its interpretation appropriate?**

Partly

- **Are all the source data underlying the results available to ensure full reproducibility?**

No source data required

- **Are the conclusions drawn adequately supported by the results?**

Yes

 Competing Interests

*No competing interests were disclosed.*

 Reviewer Expertise

 Anaemia and Iron Deficiency Anaemia in pregnancy and early childhood, Nutrition and Infectious Diseases, Nutritional epidemiology, Public Health Nutrition.

**I confirm that I have read this submission and believe that I have an appropriate level of expertise to confirm that it is of an acceptable scientific standard, however I have significant reservations, as outlined above.**

*We very much appreciate Reviewer 2’s comments and we believe the paper is* *better as a result of the changes that we have made.* *We have made significant changes to the text* *in the discussion section* *to reduce* *the* *assumption* *that we are ascribing causality which is far beyond the scope of this study.* *Further, the limitations section is expanded, and we have edited the text to ensure the reader understands the limitations of the data. We* *, however, remain confident* *, that the key points of* *this article* *are still entirely valid* *.* *Most importantly, the reviewer has helped in spotting areas requiring edits, made some suggestions as possible solution to the problems encountered by pour participants and also reminded us of the study limitations.*

# Peer review: Reviewer response for version 1

**DOI:** 10.21956/wellcomeopenres.18414.r43773

**Published:** 2021-05-28

**Authors:** Katherine L. Dickin

**Affiliations:** 1 Master of Public Health Program, Population Medicine and Diagnostic Sciences, Cornell University, Ithaca, NY, USA

The purpose of this research was to identify possible determinants of IFA supplementation adherence during pregnancy at a hospital in Kenya. The study used mixed methods, surveying mothers postnatally and interviewing health care providers on factors reported to influence adherence. This study adds to a body of literature documenting barriers to IFA supplementation across contexts and identifies implications for programs to improve nutritional care during pregnancy. The paper is well-written and clear. Concerns include the lack of clarity on how the primary outcome was defined and collected and the analytic choice to create binary variables, as well as the tendency to ascribe causal relationships to associations.

**Specific comments**

**Introduction**

 Importantly, the introduction acknowledged multiple factors that contribute to poor “adherence” since this term can imply a lack of cooperation. The authors note other barriers such as stock-outs at health facilities as such their use of “adherence” reflects more than just behavioral lapses on part of women.

**Methods**

1. The recall period of up to 6 months for postnatal mothers reporting IFA intake sees long. What evidence is there that asking so long after pregnancy did not affect the data? The paper states that many were interviewed within 3 months of delivery, but it would be helpful to clarify how many responded at various intervals and whether any differences were found in data across these time points periods. Appropriately, the possibility of recall bias acknowledged under “study limitations”.
2. A strength of this paper is that the survey data was complemented with key informant interviews (KIIs) with health care providers. In the future, it would be useful to include KIIs with mothers to gather their in-depth perspectives on barriers and facilitators of supplementation. 
3. I am not clear on the specifics of how the primary outcome was determined and the questionnaire does not appear to be available. The paper states “adherence to IFA supplementation was established by asking the participants about the average number of IFA tablets taken per week throughout the gestation period.” Was the question open-ended or multiple choice? Were mothers asked about the number of weeks or was it assumed to be “throughout gestation”? Since the majority of women attended ANC after 8 weeks, and likely quite late in pregnancy for some, how was this handled when asking about adherence “throughout gestation”? How did mothers estimate average intake per week? What was done to guard against social desirability, i.e. mothers answering as they believed was expected of them?
4. The definition of adherence as “at least five IFA tablets per week throughout gestation period” is quite a high bar, given evidence of the effectiveness of even intermittent supplementation and reports that women may adhere better when first experiencing resolution of fatigue and other anemia symptoms or in early stages of supplementation. Consider analyzing adherence as a continuous variable, or at least with multiple levels of adherence. 
5. The same concern applies to other variables such as knowledge, attitudes about providers, and even the number of ANC visits attained by the mothers. Converting the data to binary variables results in a loss of information (See Altman *et al.* BMJ. 2006 *The cost of dichotomising continuous variables* https://www.ncbi.nlm.nih.gov/pmc/articles/PMC1458573/) ^1^ and reduces the power of the analysis. It also makes results hard to interpret, since no evidence is presented on the basis for the cut points, making them appear rather arbitrary.

**Results**

1. In Table 3, it seems adherence was considered in increments (e.g. 0-2 and 3-4 and 5-7 tabs/day) but without statistical testing of differences. There is also a lack of statistical testing reported on side effects and other compliant/non-compliant comparisons. For example: “A total of 123 (51%) mothers experienced IFA related side effects. Out of this, almost half (48.0%) were non-compliant. The most prevalent side effects were vomiting (41.3%), nausea (26.9%) and dizziness (18.6%). Slightly over half (52.2%) of the mothers who experienced vomiting while on IFA were non-compliant.” There is inadequate analysis provided to support the statement in the discussion that “Participants who experienced IFA related side effects were less likely to be IFA compliant.”
2. The study includes helpful findings on factors related to potential barriers such as distance from the facility. It is notable that most mothers had a high level of knowledge of anemia. Given the concern about stockouts, it would have been good to know how often women were given IFA.
3. There are important program implications from the finding that “mothers who received education on IFA were almost three times more likely to be IFA compliant as compared to those who did not receive education on IFA (OR=2.728, 95% CI: 1.297, 5.921; p=0.009)”. Women’s desire for clear information and counseling on antenatal supplementation has been reported elsewhere (e.g. *Ethiopian women's perspectives on antenatal care and iron‐folic acid supplementation: Insights for translating global antenatal calcium guidelines into practice. *Birhanu, *et al.* Maternal & child nutrition 2018 https://onlinelibrary.wiley.com/doi/pdf/10.1111/mcn.12424) ^2^
4. Caution is needed in interpreting associations. The paper suggests causality in the statement that “attending pregnancy counselling sessions prior to conception played a significant role on IFA adherence” but this could be due to confounding if, for example, women who are very health-oriented or who have concerns due to reproductive history are more likely to seek counseling prior to pregnancy and also to be more adherent to supplementation.

**Discussion**

1. While it is useful to compare results to what has been reported in other papers, not all cited papers define compliance in the same way, limiting interpretation. Adherence definitions could be responsible for differences attributed by the authors to accessibility of the hospital, etc. For example, a common definition that was used in the cited Ethiopian and Tanzanian studies is 90 days of consumption, whereas the compliance definition in this analysis would have been over 125.
2. Some interpretations assumed that correlations were causal. There is likely to be collinearity of multiple variables with the number of ANC visits (which provide more info, more supplements, potentially more support, and indicate time and access to services), for example. Associations explored in this paper are a step toward understanding supplementation but there is a need to recognize underlying factors related to women’s mental health, autonomy, workloads, mobility, and social support, as well as quality and accessibility of health services.
3. **Study limitations: **consider also noting the risk of social desirability of responses, given quite high levels of reported adherence, and the difficulty in estimating “on average” intake. Also the limits on drawing conclusions about causality from the associations found in this analysis.

**Conclusions** are reasonable, despite some over-interpretation of causality in the discussion.

Is the work clearly and accurately presented and does it cite the current literature?

Yes

If applicable, is the statistical analysis and its interpretation appropriate?

Partly

Are all the source data underlying the results available to ensure full reproducibility?

No source data required

Is the study design appropriate and is the work technically sound?

Yes

Are the conclusions drawn adequately supported by the results?

Partly

Are sufficient details of methods and analysis provided to allow replication by others?

Partly

Reviewer Expertise:

Maternal and child nutrition, social and behavior change, qualitative and mixed methods research, nutrition program implementation and effectiveness

I confirm that I have read this submission and believe that I have an appropriate level of expertise to confirm that it is of an acceptable scientific standard, however I have significant reservations, as outlined above.

## References

[1] : The cost of dichotomising continuous variables.*BMJ*.2006;332(7549) : 10.1136/bmj.332.7549.1080108010.1136/bmj.332.7549.108016675816PMC1458573

[2] : Ethiopian women's perspectives on antenatal care and iron-folic acid supplementation: Insights for translating global antenatal calcium guidelines into practice.*Matern Child Nutr*.14 Suppl 1: 10.1111/mcn.1242410.1111/mcn.1242429493899PMC6866194

# Response

**Published:** 2021-06-29

**Authors:** Felix Bahati

**Affiliations:** KEMRI WELLCOME TRUST, Kenya

The purpose of this research was to identify possible determinants of IFA supplementation adherence during pregnancy at a hospital in Kenya. The study used mixed methods, surveying mothers postnatally and interviewing health care providers on factors reported to influence adherence. This study adds to a body of literature documenting barriers to IFA supplementation across contexts and identifies implications for programs to improve nutritional care during pregnancy. The paper is well-written and clear. Concerns include the lack of clarity on how the primary outcome was defined and collected and the analytic choice to create binary variables, as well as the tendency to ascribe causal relationships to associations.

**Specific comments**

**Introduction**

 Importantly, the introduction acknowledged multiple factors that contribute to poor “adherence” since this term can imply a lack of cooperation. The authors note other barriers such as stock-outs at health facilities as such their use of “adherence” reflects more than just behavioral lapses on part of women.

*We appreciate that we had used the term ‘adherence’* *to imply that mothers are not taking the supplements as required without acknowledging the fact that some of the reasons behind such non-adherence trends are way beyond their control.  A good example is the IFA stock-out within facilities as pointed out by the reviewer, something that these mothers may not have control about. We have therefore edited the fourth paragraph of our introduction to include a statement clearly stating that some of these causes of poor adherence are beyond the control of these women.*

**Methods**

1. The recall period of up to 6 months for postnatal mothers reporting IFA intake sees long. What evidence is there that asking so long after pregnancy did not affect the data? The paper states that many were interviewed within 3 months of delivery, but it would be helpful to clarify how many responded at various intervals and whether any differences were found in data across these time points periods. Appropriately, the possibility of recall bias acknowledged under “study limitations”.

*We have added a whole paragraph in the discussion section to address the recall bias issue. We have cited evidence supporting our recall period of 0-6 months. Generally, pregnancy is a very important event in the lifetime of most women. According to other researchers, when an event is salient, the respondents tend to remember it even 20 months afterwards. This coupled with overall assessment of an event i.e asking the respondent to gauge their overall use of IFA in gestation rather than specific weeks/stages of gestation makes it easier for participants to remember. We are therefore confident that the findings reported in this article are relevant and were not influenced by recall bias to a great extent. We have also edited table 2 and provided the total number of respondents that we interviewed at every monthly interval post-delivery as suggested by the reviewer. The limitation section has also been updated to clearly show the recall period was a weakness in this study.*

1. A strength of this paper is that the survey data was complemented with key informant interviews (KIIs) with health care providers. In the future, it would be useful to include KIIs with mothers to gather their in-depth perspectives on barriers and facilitators of supplementation. 

*We thank the reviewer for this useful direction. This is a good suggestion that could help us unmask more barriers and facilitators of IFA supplementation that we may not have achieved with the semi-structured questionnaire that was used in the current study. We shall consider this in our future studies.*

  

1. I am not clear on the specifics of how the primary outcome was determined and the questionnaire does not appear to be available. The paper states “adherence to IFA supplementation was established by asking the participants about the average number of IFA tablets taken per week throughout the gestation period.” Was the question open-ended or multiple choice? Were mothers asked about the number of weeks or was it assumed to be “throughout gestation”? Since the majority of women attended ANC after 8 weeks, and likely quite late in pregnancy for some, how was this handled when asking about adherence “throughout gestation”? How did mothers estimate average intake per week? What was done to guard against social desirability, i.e. mothers answering as they believed was expected of them?

*We measured adherence to IFA supplementation during pregnancy using a set of two questions. Firstly, we asked the respondent whether they took the IFA tablets faithfully throughout pregnancy as recommended by the healthcare provider, “* *Did you take the IFA supplements faithfully as recommended by the health care provider?* *(Yes/No)”.* *This was confirmed further by asking about the average number of IFA tablets* *that the respondents consumed per week throughout the gestation, “On average, how many IFA tablets did you used to take every week throughout pregnancy? (open ended).” Although we had assumed taking the IFA tablets faithfully as recommended to mean taking IFA daily as WHO recommends, we noticed that some respondents who reported taking the tablets faithfully also reported taking as low as three IFA tablets per week on average. For the sake of standardization of our primary outcome, we defined adherence as having taken at least five IFA tablets per week throughout pregnancy (Similar to having used the supplements for an average of at least 5 days per week throughout the gestation). Although the respondents were classified as either adherent or non-adherent based on the number of tablets consumed, we did not classify respondents who started ANC less than 3 months due to delivery as compliant. This was because we reasoned that they had consumed the supplements for a shorter duration compared to others. Nonetheless, the authors acknowledge that by measuring adherence throughout gestation without specifying weeks, we failed to establish a specific interval over which adherence was measured. We have further acknowledged this in the limitation section. All in all, it would have been difficult for the respondents to remember their IFA use patterns had we specified some gestation weeks. We avoided instances where the participants could provide inaccurate responses for social desirability by: 1) Explaining the justification of the study that we only intended to use the data to influence policies meant to improve IFA uptake in pregnancy and avoid pregnancy complications and 2). Assuring the participants of their confidentiality such that they were not going to be victimised in any way based on their responses. Further, we sought permission from the hospital management to allow our interviewer to interact with the mothers within the clinics without wearing a medical regalia such as a white coat to avoid creating any impression that he was a healthcare provider consequently risking responses of social desirability.  *

1. The definition of adherence as “at least five IFA tablets per week throughout gestation period” is quite a high bar, given evidence of the effectiveness of even intermittent supplementation and reports that women may adhere better when first experiencing resolution of fatigue and other anemia symptoms or in early stages of supplementation. Consider analyzing adherence as a continuous variable, or at least with multiple levels of adherence. 

*We understand the point that the reviewer is trying to put across here. Taking an average of five tablets of IFA per week may seem quite higher.* *We have added a small section in the discussion section to highlight this and make our readers aware. We realized that we based much of this study on the current WHO guidelines of IFA supplementation and tried as much as possible to stick to their recommendation of adherence as daily intake of IFA right from conception to birth. This might have made us set the bar quite high. Our reasoning was also informed by other studies conducted in other parts of Kenya where more than 4 IFA tablets per week were used as a cut off for adherence. Despite such studies having focused a specific week among ANC seeking mothers, the fact remains that the assumption is that the mothers will stick to the same trend throughout pregnancy. We are confident that our findings suit well with the current WHO IFA supplementation guidelines and can easily inform policies within this context. The reviewer’s preference on the use of adherence as a continuous variable is a keen observation and justified. We overlooked this during our analysis, and fully concur with the reviewer that it would have been better to use adherence as a continuous variable or establish various levels of adherence. This is a weakness in our data analysis approaches and we have stated it in the limitations. We believe that our table 3 highlights some various levels of adherence to some extent.*

1. The same concern applies to other variables such as knowledge, attitudes about providers, and even the number of ANC visits attained by the mothers. Converting the data to binary variables results in a loss of information (See Altman *et al.* BMJ. 2006 *The cost of dichotomising continuous variables* https://www.ncbi.nlm.nih.gov/pmc/articles/PMC1458573/) ^1^ and reduces the power of the analysis. It also makes results hard to interpret, since no evidence is presented on the basis for the cut points, making them appear rather arbitrary.

*Actually, the reviewer made us realize that by converting the continuous variables into binary we sacrificed clarity and the statistical rigor in favour of brevity. As already stated above, we have discussed this as a limitation in the limitation section.*

**Results**

1. In Table 3, it seems adherence was considered in increments (e.g. 0-2 and 3-4 and 5-7 tabs/day) but without statistical testing of differences. There is also a lack of statistical testing reported on side effects and other compliant/non-compliant comparisons. For example: “A total of 123 (51%) mothers experienced IFA related side effects. Out of this, almost half (48.0%) were non-compliant. The most prevalent side effects were vomiting (41.3%), nausea (26.9%) and dizziness (18.6%). Slightly over half (52.2%) of the mothers who experienced vomiting while on IFA were non-compliant.” There is inadequate analysis provided to support the statement in the discussion that “Participants who experienced IFA related side effects were less likely to be IFA compliant.”

*“* *Participants who experienced IFA related side effects were less likely to be IFA compliant* *’ was based on the odds ratios obtained from the multivariable logistic regression presented in table 6 and not the descriptive statistics as the reviewer depicts.*

1. The study includes helpful findings on factors related to potential barriers such as distance from the facility. It is notable that most mothers had a high level of knowledge of anemia. Given the concern about stockouts, it would have been good to know how often women were given IFA.

- *We did not collect this information directly as stated by the reviewer. This information was collected indirectly by asking the health care providers about IFA stock-outs.  As one of the health care providers noted during the KII, “… As I said, we need to have a nutritionist in every room but that’s almost impossible for now. So sometimes when I’m compiling monthly reports, I notice that the mothers have not been issued with the supplements for some time. When you make follow-ups, you will be told that IFA had run out of stock. But the problem is that they did not communicate because I could have looked for the supplements somewhere else even if it means borrowing from another facility…” (KII: healthcare provider). We also asked the mothers whether there was a time that they visited ANC and were not issued with supplements due to stock outs. Only 12 % (n=29) of the mothers said that they failed to get IFA tablets during ANC due to stock outs of the commodity at the facility. In almost all the 12% of the cases, this only happened once. We have included the sentences below in the results section to show how often the participants were given the supplements. “*Only 12% of the participants interviewed reported not having been given IFA supplements during ANC visits because the facility had run out of stock. However, this only happened once in almost all the 12% of the cases. Separately, KIIs with healthcare workers also revealed that the facility runs out of the supplements at times, although not very often.”

1. There are important program implications from the finding that “mothers who received education on IFA were almost three times more likely to be IFA compliant as compared to those who did not receive education on IFA (OR=2.728, 95% CI: 1.297, 5.921; p=0.009)”. Women’s desire for clear information and counseling on antenatal supplementation has been reported elsewhere (e.g. *Ethiopian women's perspectives on antenatal care and iron* *‐* *folic acid supplementation: Insights for translating global antenatal calcium guidelines into practice.* **Birhanu, *et al.* Maternal & child nutrition 2018 https://onlinelibrary.wiley.com/doi/pdf/10.1111/mcn.12424) ^2^

*We have read the suggested work and even cited it in the discussion (ref no..) to make our discussion even better.*

1. Caution is needed in interpreting associations. The paper suggests causality in the statement that “attending pregnancy counselling sessions prior to conception played a significant role on IFA adherence” but this could be due to confounding if, for example, women who are very health-oriented or who have concerns due to reproductive history are more likely to seek counseling prior to pregnancy and also to be more adherent to supplementation.

*We acknowledge that the statement highlighted by the reviewer could easily be misinterpreted to mean that we are ascribing ANC attendance to causality which is wrong. We have rephrased the sentence to imply an association between ANC counselling and adherence.*

**Discussion**

1. While it is useful to compare results to what has been reported in other papers, not all cited papers define compliance in the same way, limiting interpretation. Adherence definitions could be responsible for differences attributed by the authors to accessibility of the hospital, etc. For example, a common definition that was used in the cited Ethiopian and Tanzanian studies is 90 days of consumption, whereas the compliance definition in this analysis would have been over 125.

*The reviewer’s* *thought on the possible reason for the disparities in IFA adherence prevalence between our study and other similar studies conducted elsewhere* *is indeed more apt than* *what we thought could be due to hospital accessibility. We have therefore re-adjusted the first paragraph to include the reviewer’s thought.*
* *

1. Some interpretations assumed that correlations were causal. There is likely to be collinearity of multiple variables with the number of ANC visits (which provide more info, more supplements, potentially more support, and indicate time and access to services), for example. Associations explored in this paper are a step toward understanding supplementation but there is a need to recognize underlying factors related to women’s mental health, autonomy, workloads, mobility, and social support, as well as quality and accessibility of health services.

*The scope of this study was to establish the associations and not to ascribe causality. We have therefore rephrased all the sentences that seemed to portray causality rather than association. Besides, we have provided a section in the discussion clearly stating that the association revealed in this study are subject to collinearity as elaborated by the reviewer.*


* *

1. **Study limitations: **consider also noting the risk of social desirability of responses, given quite high levels of reported adherence, and the difficulty in estimating “on average” intake. Also the limits on drawing conclusions about causality from the associations found in this analysis.

*All the observation raised here by the reviewer have been considered partly in the discussion and fully in the limitation section.*

**Conclusions** are reasonable, despite some over-interpretation of causality in the discussion.

- **Is the work clearly and accurately presented and does it cite the current literature?**

Yes

- **Is the study design appropriate and is the work technically sound?**

Yes

- **Are sufficient details of methods and analysis provided to allow replication by others?**

Partly

- **If applicable, is the statistical analysis and its interpretation appropriate?**

Partly

- **Are all the source data underlying the results available to ensure full reproducibility?**

No source data required

- **Are the conclusions drawn adequately supported by the results?**

Partly

*Generally, w* *e very much appreciate Reviewer* *1* *’s comments and we believe the paper is stronger and more precise as* *a* *result of the changes we have made in response. We have made significant changes to the text to reduce assumptions* */causality misinterpretations* *and instead speak to the data we have. Further, the limitations section is expanded, and we have edited the text to ensure the reader understands the limitations of the* *study* *. We believe, however, that the key points of the text are still entirely valid* *.*
